# Zika, abortion and health emergencies: a review of contemporary debates

**DOI:** 10.1186/s12992-019-0489-3

**Published:** 2019-07-24

**Authors:** Clare Wenham, Amaral Arevalo, Ernestina Coast, Sonia Corrêa, Katherine Cuellar, Tiziana Leone, Sandra Valongueiro

**Affiliations:** 1 Department of Health Policy, London School of Economics and Political Science, Houghton Street, London, WC2A 2AE UK; 2 Instituto de Medicina Social-Universidade do Estado do Rio de Janeiro, Rua São Francisco Xavier 524, Maracanã, Bloco D, 7° andar, UERJ, Rio de Janeiro, RJ 20550-013 Brazil; 3 Department of International Development, London School of Economics and Political Science, Houghton Street, London, WC2A 2AE UK; 4 ABIA, Sexuality Policy Watch, Avenida Presidente Vargas, 446, 13th floor, CEP 20071-907, Rio de Janeiro, RJ Brazil; 5 National Faculty of Public Health, Universidad de Antioquia, Calle 62 #52-59, Medellín, Colombia; 6 Post-graduate Programe of Public Health, Federal University of Pernambuco, Av Prof Moraes Rego, s/n. Hospital das Clínicas, Bloco “E” – 4° Andar. Cidade Universitária CEP: 50.670-901, Recife, PE Brazil

**Keywords:** Zika, Abortion, Health emergencies, Sexual and reproductive health, Structural violence, Reproductive rights

## Abstract

**Background:**

The Zika outbreak provides pertinent case study for considering the impact of health emergencies on abortion decision-making and/or for positioning abortion in global health security debates.

**Main body:**

This paper provides a baseline of contemporary debates taking place in the intersection of two key health policy areas, and seeks to understand how health emergency preparedness frameworks and the broader global health security infrastructure is prepared to respond to future crises which implicate sexual and reproductive rights. Our paper suggests there are three key themes that emerge from the literature; 1) the lack of consideration of sexual and reproductive health (SRH) services in outbreak response 2) structural inequalities permeate the landscape of health emergencies, epitomised by Zika, and 3) the need for rights based approaches to health.

**Conclusion:**

Global health security planning and response should specifically include programmatic activity for SRH provision during health emergencies.

## Introduction and background

We set out to understand the intersection of health emergencies and abortion, using the case study of the Zika outbreak (2015–7). We chose to examine this intersection in Brazil, Colombia and El Salvador, each of these countries had Zika infection (albeit with different incidence), yet represent different regulatory environments for abortion, ranging from legalisation in Colombia [1] to criminalisation in El Salvador [2] and Brazil, which even lists abortifacient medication on its prohibited drugs list [3]. During the Zika outbreak requests for online abortion pills from women in Brazil doubled between November 2015 and March 2016, and increased by more than a third in El Salvador (35%) [4] and in Colombia (38.7%) [5]. Whilst these findings cannot be conclusively attributed to the Zika outbreak, it led us to question how health emergencies and abortion inter-relate in practice, and what implications this might have for health emergency preparedness for the future.

We suggest that the Zika outbreak provides the empirical setting for considering the impact of health emergencies on abortion decision-making and/or for positioning abortion in global health security debates. In doing so, we establish a baseline of contemporary research raised by this intersection of health policy areas, and seek to understand how health emergency preparedness frameworks and the broader global health security infrastructure is prepared to respond to future crises which implicate sexual and reproductive rights. Our commentary suggests there are three key themes that emerge from the literature; 1) the lack of consideration of sexual and reproductive health (SRH) services in outbreak response 2) structural inequalities permeate the landscape of health emergencies, and 3) the need for rights based approaches to health.

Before presenting these three key findings, we will first define our three areas of investigation.

### Health emergencies

Health emergencies fit within a broader framework of global health security [6, 7] and refer to acute public health events which the World Health Organization (WHO) defines as “an extraordinary event which … constitute [s] a public health risk to other states through the international spread of disease and/or to potentially require a coordinated response” [8]. This has been embodied in legislation through the WHO’s Public Health Emergency of International Concern (PHEIC) and mirrored in national legislation such as Brazil’s “*Emergência em Saúde Pública de Importância Nacional*” (Public Health Emergency of National Importance) (ESPIN). Such policies are important to analyse as the designation of an emergency response allows response activities which go beyond routine health control measures, often focused on short-term outcomes to end an outbreak event. These can include extraordinary financing, restrictive quarantine measures or calling on non-routine health providers, such as the military, to provide assistance. Importantly, such policies promote a firefighting approach to disease control rather than systemic changes that may provide more sustainable long-term capacity to manage or minimise future outbreaks and build more resilient health systems.

### Zika

Zika is an aborviral disease spread by the *Aedes aegypti* mosquito. For most people the infection only displays minor flu-like symptoms and has no lasting consequences. However, during 2015–6, assertions were made by clinicians and researchers in Brazil concerned by a cohort of women who were infected with Zika (or Zika-like symptoms) during their pregnancy and went on to have babies born with microcephaly (a neonatal malformation where a baby’s head is smaller than expected) and other developmental complications collectively called Congenital Zika Syndrome (CZS). In Brazil, this led to an “emergencised” response, including the designation of an ESPIN and the deployment of the military to combat the mosquito.

On 1st February 2016, the WHO declared Zika related microcephaly to be a PHEIC. Notably, this PHEIC was declared “not on the basis of what is currently known about the Zika virus …. rather on the basis of what is not known about the clusters of microcephaly” [9]. Further research has proved a causal relationship between Zika virus infection during pregnancy and CZS [10, 11]. It is estimated that between 5 and 15% of babies whose mothers are known to have had a Zika infection whilst pregnant are infected with CZS [12–15].

The key traits of the securitized response to this outbreak were 1) destruction and fumigation of mosquitos and their breeding grounds, 2) a medicalized focus on development of vaccines, diagnostics and genetically modified mosquitos [16], 3) advising women of reproductive age that they should not get pregnant [17]. Whilst this policy response (alongside the epidemic characteristics of the disease reducing the susceptible population) resulted in a sharp decline in new cases of Zika (and CZS), what was notably absent from the response was access to abortion options.

### Abortion

Abortion is a common feature of people’s reproductive lives. While procedures for inducing safe abortion are straightforward – including medical abortion, the use of a drug or combination of drugs for pregnancy termination - whether or not abortion is available or un/safe is influenced by a complex mix of politics, access, social attitudes and individual experiences [18]. Importantly, two countries in Latin America that had high incidences of Zika during the 2015–7 outbreak have some of the most restrictive abortion regulations globally:

Abortion is illegal in Brazil, except when a pregnancy may threaten a woman’s life or if it is the result of a case of rape or incest (article 128, I and II) [19]. In 2012, the Brazilian Supreme Court voted in favour of allowing abortion in the case of anencephaly (where a foetus is developing without a brain) [20]. In El Salvador, abortion is illegal and criminalized, according to Chapter II of the Salvadorian Penal Code [21]. As a consequence, dozens of El Salvadorean women are currently serving prison sentences of up to 40 years on abortion charges, even if it is unclear if they suffered a spontaneous (miscarriage) or induced abortion [22].

Despite current regulatory restrictions in Brazil, the National Survey on Abortion (PNA) observed that clandestine abortion is a common practice among women [23]. By the age of 40, one in five Brazilian women have had an abortion via different methods dependent on socio-economic status, including homeopathic treatment and medical abortion for lower socio-economic  groups and private medical and surgical solutions for higher wealth groups [24]. Rates are higher in the North/Northeast region, higher among poorer populations, and higher among women of colour [23].

Conversely, Colombia has some of the most progressive abortion laws regionally, with abortion permitted when the pregnancy threatens the woman’s health (broadly defined to include mental and physical concerns); when the pregnancy is a result of rape, incest or artificial insemination without consent; when fetal malformations incompatible with life are diagnosed. Interestingly the legislation does not specify a gestational deadline for abortion, and later term abortions can be sought where they may cause severe anguish or mental disorders [1].

An estimated 56 million induced abortions occur globally each year [25] of which 54.9% are unsafe^1^ [26]. This results in a major public health problem, especially in contexts where access to legal abortion is highly restricted. An estimated 7.9% of maternal deaths are due to unsafe abortion [27]. In the aforementioned Zika affected countries this is evident, with approximately 250,000 emergency room visits resulting from complications from unsafe abortions annually [28]. Even in Colombia where regulations permit abortion, there are significant barriers for women’s access [29] and a significant number of unsafe abortions continue [30].

### Zika, abortion and health emergencies

The Zika outbreak brings to the fore the need to integrate comprehensive SRH, including safe abortion, as an important component of the response to the health emergency, yet this has not occurred. The growing importance of SRH in crisis settings has been recognised, including the importance of access to contraception and maternal care [31]. Work by McGinn and Casey [32] has put the discussion of abortion in humanitarian settings on the agenda, but none of this work has considered health emergency as a crisis zone for such analysis. Understanding the complexity around obtaining abortion-related care in the context of Zika is urgently needed, especially in light of the intense attention abortion receives, owing to shifting national and international laws, policies, treaties, protocols and funding provision [33]. This commentary represents the first step of a larger project exploring this unmet need for abortion during health emergencies, through desk-based research and convening a workshop with leading academics and activists working across the domains of Zika, health emergencies and abortion. In doing so, we have identified three areas where we can see the intersection between these pivotal health policy issues:

1. *SRH only partially woven into the Zika response*

A key trait witnessed in the policy response by multiple governments to Zika was to recommend that women avoid or delay pregnancy [34–37]. Encouraging contraception was one mechanism by which governments promoted such a policy, although it was unclear how women might access these contraceptives in all settings [38]. In Brazil and Colombia it was further advised that pregnant women should not travel to endemic areas, and that those living in these areas should take precautions to avoid mosquito bites [34, 39]. To some extent, this advice was heeded; 56% of Brazilian women said they had either avoided or tried to prevent a pregnancy because of the health emergency [40], yet this decision was affected by education status, age, religion, socio-economic status, and access to healthcare services [41]. Moreover, a decline of live births subsequent to the Zika outbreak, suggests that women must have taken some steps to avoid or terminate pregnancy [24, 42]. Importantly, however, restrictions on abortion rule out strategies based on screening for CZS infection, which offer the potential to limit the consequences of the virus, but not fertility.

Despite these statistics, one of the most concerning aspects of these governments’ Zika response protocols was the absence of reference to abortion [43]. Only Colombia’s Zika policy listed information about legal abortion practices [44, 45]. However, this advice may not have been widely publicised, with Zika infected Colombians stating that they were never provided with any information about termination of pregnancy as part of their care [46]. This comprehensive information about access to contraception and education around legal reproductive choices must be provided during health emergencies. Zika has shown that a health emergency can have externalities that affect women’s reproductive lives and informed options must be available to them, even in abortion restricted settings.

2. *Responsibility and Structural Inequalities*

Policies that advise women avoid pregnancy, use contraception, wear long sleeves, or use insecticide imply that it is the individual woman’s choice, and therefore responsibility to self-manage her risk profile during the outbreak. Mirroring criticisms of policies which placed women in a position of responsibility for HIV/AIDS prevention [47], taking heed of government positions for Zika might imply that if a woman does have a baby with CZS then it is the woman’s responsibility for not following official advice [48]. Such policies fail to take into account the structural inequalities that pervades everyday life in Latin America. Nunes and Pimenta have established structural factors which put traditionally neglected communities at higher risk of being bitten by a mosquito [49]. This includes poor, black or indigenous women, living in remote locations, with low levels of education dominated by the rampant inequalities that pervade Latin American society [50, 51]. Velez and Diniz consider Zika to be a hidden pandemic [37] as it is women, without political capital, who have ultimately born the burden of raising children with CZS and the least financially equipped to access medical and social support that a child with a disability needs [52].

Moreover, by placing responsibility onto women, it suggests that men do not have the same responsibility for pregnancy and further implications of CZS, and creates an unequal gender dynamic to the outbreak [51]. Davies and Bennett highlight the gaps rendered by those in the public health community responding to an outbreak of infectious disease which ignore the structural gender inequalities which pervade most health systems. They refer to this in relation to Watson and Mason’s “tyranny of the urgent” which relegates structural concerns to low priority in order to exercise the autonomy presumed by the international advice (in this case, the declaration of the PHEIC) [53].

Further, Moeller argues that by framing Zika infection control as a woman’s responsibility, governments may be able to downplay their own inadequacies and failures in limiting the spread of the mosquito through improved water and sanitation facilities or the provision of sexual and reproductive health services [54]. In the northeast of Brazil, where the epicentre of CZS cases occurred, only 51% of households had access to basic sanitation in 2012 [55]. This means that households are required to collect and store water in houses, providing a fertile reservoir for vector development that is inside homes and close to humans [56–58]. Souza et al. showe that people with precarious living conditions had a higher prevalence of CZS compared with those who live in better living conditions [59].

We know from the Ebola outbreak that the crisis exposed weaknesses across health systems [60, 61]. Similarly, the Zika outbreak demonstrated the strength in the health system, particularly in Brazil for rapid vector control activity and sophisticated biomedical research on the virus, transmission patterns and pathogenic effects on foetuses and babies. In contrast, it revealed a systematic gap in women’s health promotion and provision. Previous research demonstrates that Zika hit an already overstretched reproductive health system [62–65], with high rates of unintended pregnancies in the region, and questions of access to (affordable) contraceptives or clandestine abortions [66].

These structural factors are not dissimilar to other infectious disease outbreaks, which tend to occur because of environmental factors including deforestation, precarious urbanisation, overcrowded sub-sufficient housing etc. [37]. It is well established that pandemics disproportionately affect the most disadvantaged, meaning that neutral approaches to global Zika planning and response, such as resource allocation will perpetuate and increase existing gender, racial, social and health disparities [53, 67]. As such, responses to health emergencies must actively engage with mechanisms to overcome these barriers, to find a meaningful way to ensure that those most affected or at risk of an outbreak have access to health care and suitable resources when needed.

3. *Rights based approaches to health emergencies*

Zika underscores the importance of engaging a rights based frame to situate abortion and reproductive health services during health emergencies. Such championing of a rights based approach is not new in public health emergencies [53] [68], and the vulnerability of certain groups has been recognised in the WHO Emergency Response Framework [69]. However, it is even more apparent in the Zika outbreak when considering intersecting issues of SRH, gender, access to health services, as well as in locations with entrenched histories based on rights based approaches to health. Several critiques of Zika policy responses have focused in on issues of women’s ownership and self-determination of their bodies, based on CEDAW’s General Recommendation (No. 24) [40, 70–72]. The WHO echoed this rights based approach encouraging policies that allow women and girls to make their own decisions about pregnancy and childbirth [48], although constrained by the sovereign legislation in member states.

For example, in El Salvador, it has been argued that national laws penalizing abortion during the Zika outbreak (and beyond) do not comply with human rights or international law [48]. Activists have demanded the Salvadorian government take a more rights based approach to the Zika outbreak through legislative reforms to decriminalize abortion [73]. In 2017 (during the Zika outbreak), the UN High Commissioner for Human Rights asked for a moratorium on the criminalisation of women for having an abortion in El Salvador, suggesting that this contradicts international human rights obligations [46]. However, the implementation of a rights based approach faces many barriers. One of them is technocratic resistance to incorporate a consistent rights frame in response to health emergencies. A second, and often more stringent, are anti-abortion views and their ideological effect in health emergencies. In the case of Brazil, for example, groups contesting abortion rights have argued that prioritising womens' right to pregnancy termination in the Zika outbreak  violates the rights of disabled babies. There are also those who raised concerns that by focusing too much on the contentious issue of reproductive rights, this can distract from bigger structural factors with wider reaching benefits [65].

## Conclusion

Considering these intersecting themes of Zika, health emergencies and abortion we have centred on three key issues – the role of SRH in health emergencies, structural inequalities and rights based approaches to health [74]. Cutting across these themes is the need for an explicitly gendered or feminist approach to better understanding the connections between Zika, health emergencies and abortion.

However, beyond the literature, we recognise that the Zika crisis and the crisis women find themselves in when seeking an abortion in restrictive settings are not independent of each other. The precarious position women faced during the Zika outbreak, without routine access to SRH services or reproductive rights, whether due to regulation or structural factors, can focus discussions around the depenalisation and liberalisation of abortion laws.

In 2016, the Brazilian National Association of Public Defenders (with support from NGO Anis-Institute of Bioethics) petitioned the Brazilian Supreme Court to allow abortion in the case of women infected by Zika (ADI 5581) [75]. This was followed by the Partido Socialismo e Liberdade (PSOL) (ADPF 442) calling for a full decriminalisation of abortion up to 12 weeks, and a public hearing in the Supreme Court seeking multi-sectoral opinions on matter was held in August, 2018 [76]. The central argument to the first claim is the state’s failure to control the vector, and a woman’s dignity and health, considering her social and economic vulnerability [77]. The ADPF 442 is still pending in the Supreme Court at the time of writing, yet the fact that this occurred shows the catalytic effect that health emergencies can have on regulatory development.

We suggest that behavioural recommendations, such as avoiding pregnancy reinforce unequal power relationships between women and the government with regards to reproductive health options, and instead that health emergency preparedness activity including national response plans, and the International Health Regulations (IHR 2005) should move beyond these behavioural considerations and engage with more gender sensitive policies and specifically include programmatic activity for access to SRH provision during health emergencies [74].

## Data Availability

Data sharing is not applicable to this article as no datasets were generated or analysed during the current study.

## Footnotes

1 An **unsafe abortion** is the termination of a pregnancy by people lacking the necessary skills, or in an environment lacking minimal medical standards, or both.
